# Biosynthesis and characterisation of solid lipid nanoparticles and investigation of toxicity against breast cancer cell line

**DOI:** 10.1049/nbt2.12062

**Published:** 2021-06-04

**Authors:** Mohammad Sharifalhoseini, Ali Es‐haghi, Gholamhassan Vaezi, Hooman Shajiee

**Affiliations:** ^1^ Department of Biology Damghan Branch Islamic Azad University Damghan Iran; ^2^ Department of Biology Mashhad Branch Islamic Azad University Mashhad Iran

## Abstract

Solid lipid nanoparticles (SLNs) comprise non‐toxic surface‐active lipidic agents combined with appropriate ratios of drugs or essential oils. The goal of this research was to investigate the effects of the SLN synthesised using essential oils of *Foeniculum vulgare* on the MCF‐7 breast cancer cell line. SLNs were prepared by homogenisation and ultrasound techniques and characterised by dynamic light scattering (DLS), zeta potential assessment, and transmission electron microscopy (TEM). 3‐(4,5‐dimethylthiazol‐2‐yl)‐2,5‐diphenyl tetrazolium bromide assay (MTT assay), flow‐cytometry, and Acridine‐Orange assay were employed for assessing the biological activities of the SLNs. The average particle size was 55.43 nm and the net surface charge was −29.54 ± 11.67 mV. TEM showed that the mean particle size was 33.55 nm and the synthesised SLNs had a uniform round morphology. The MTT assay showed that the prepared SLNs had high toxicity against MCF‐7 cells and low toxicity against normal HUVECs cells. Flow‐cytometry revealed a noteworthy rise in the subG1 peak of the cell cycle in the cancer cells treated with SLNs compared to the controls, indicating apoptosis in cancer cells. The results also showed discolouration in SLNs‐treated cells, which further confirmed the induction of apoptosis and the toxicity of the SLNs against MCF‐7 cells.

## 1 INTRODUCTION

Solid lipid nanoparticles (SLNs) were first introduced in 1991 as alternatives to conventional colloidal carriers such as emulsions and polymeric nanoparticles (NPs) [1, 2, 3]. SLNs are colloidal carriers with the sizes of 10–1000 nm and consist of biological lipids dispersed in an aquatic phase [4]. SLNs, which are solid at the body temperature, are the first generation of the lipid‐based nanocarriers formulated from lipids and stabilised by emulsifiers [5]. Solid lipids are used instead of liquid oils to overcome the problems related to the presence of fat in the aquatic phase. Active ingredients such as drugs or essential oils are first melted or blended with the lipid phase and then added to the aquatic phase [6]. The benefits of SLNs are controlled drug release, biocompatibility, sustainability, stability of pharmaceutical formulations, high loading capacity, easy sterilisation, chemical protection from active materials, and easy preparation methods [7, 8]. Therefore, SLNs can be used to carry drugs to enhance their effectiveness and stability.

Drug delivery using SLNs depends on various factors such as the administration route, the nature of lipids and active substances, cellular interactions, as well as the particles’ sizes and formulations [8, 9, 10]. Lipase is the most important enzyme that affects the structure of SLNs within the body. The rate of degradation of different lipids by this enzyme is variable [11, 12]. Lipids with longer chains have slower degradation; moreover, the presence of some emulsifiers, as lipid protectants, decreases the rate of degradation [13, 14].

In chemotherapy, cytotoxic drugs are used to induce apoptosis in cancer cells and prevent the progression of tumours by affecting the cell cycle and producing free radicals [15, 16]. SLNs, with relatively low side effects, can increase the efficiency of chemotherapeutic agents and augment their anti‐cancer properties to prevent tumour formation, angiogenesis, metastasis, and progression [17, 18]. Due to their exclusive physical and chemical characteristics, NPs‐based platforms are considered promising cancer treatment schemes [19, 20, 21, 22, 23, 24].

The biosynthesis of NPs offers a perspective to expand their applications in many fields like biosensing, photocatalysis, medicine, and food industries [25, 26, 27, 28, 29]. Recently, interest in natural products has grown due to their benefits and low side effects [30, 31, 32]. Among them, essential oils have widely been utilised in medicine as well as in pharmaceutical, food, and agronomy applications [33, 34]. In addition to their widespread usages in various industries, these oils have attracted attention because of their roles in preventing and treating different diseases [35, 36]. Using a green and safe method to fabricate different types of NPs can significantly contribute to their safety and reduce their toxicity [37, 38, 39, 40]. *Foeniculum vulgare* Mill, commonly called fennel, has been employed in traditional medicine for its widespread beneficial effects on diseases related to the lung and gastrointestinal tract as well as the endocrine and reproductive systems [41, 42]. Many studies have shown that *F. vulgare* is remarkably effective in managing several bacterial/fungal/viral diseases [43, 44].

In this study, we assessed the potential of *F. vulgare* in the synthesis of SLNs and also investigated the anti‐cancer properties of the fabricated SLNs.

## 2 MATERIALS AND METHODS

### 2.1 Materials

DMEM, FBS, trypsin, 3‐(4,5‐dimethylthiazol‐2‐yl)‐2,5‐diphenyl tetrazolium bromide (MTT) reagent, and acridine orange dye were purchased from Sigma‐Aldrich Company, Ltd. The other reagents not mentioned here were from Merck. The cancerous cell line (MCF‐7) and the human umbilical vein endothelial cells (HUVEC) were obtained from the Pasteur Institute of Iran. Transmission electron microscopy (TEM) was performed by LEO 912 AB on carbon‐coated copper grids with an accelerating voltage of 120 kV, and clear microscopic views were observed in a diverse range of magnifications. Zetasizer Pro was used to measure the dynamic light scattering (DLS) and zeta potential. For both zeta potential and particle size analyses, lipid NP suspensions were placed in a 4‐ml acrylic cuvette. Most measurements were performed at 25°C, and the solvent was double distilled water at all times.

### 2.2 Preparation of solid lipid nanoparticles

The SLNs containing essential oils were prepared using homogenisation and ultrasound methods. First, 400 mg stearic acid was added to 25 ml distilled water. In this method, the lipid and water phases were heated in a bain‐marie at 80°C. Then a phosphatidylcholine (lecithin), as a surfactant (200 mg), and *F. vulgare* essential oil (100 ml) were added to the lipid phase (stearic acid) in the bain‐marie. Thereafter, the water phase containing 1% Tween 80 and distilled water was added to the lipid phase in the bain‐marie at 80°C. To prevent the solidification of the lipid phase, it was maintained in a water bath at 80°C until it was transferred into a high‐speed homogeniser. Homogenisation was conducted at the speed of 12,000 rpm for 6 min. After that, the mixture was sonicated using an ultrasonic probe sonicator at room temperature for 10 min. The prepared SLNs were frozen, dried, and used without further purification [8].

### 2.3 Cells' maintenance

The purchased cancerous and normal cell lines (i.e. MCF‐7 and HUVEC) were grown in DMEM containing antibiotics (100 *µg*/ml penicillin and 100 UI/ml streptomycin) and FBS 10%. Incubation was performed at 5% CO_2_ and 37℃. After that, 5 × 10^3^ cells were seeded at each of the wells of a 96‐well plate and incubated for 12 h. Different SLN concentrations (0, 31.2, 62.5, 125 and 250 *µg*/ml) were used to treat the cells for 24, 48, and 72 h. Every 48 h, the fresh culture medium was replaced. After the cells reached a confluence of 75%, the trypsin enzyme (0.025% trypsin and 0.02% EDTA) was applied to detach the cells that were then washed with phosphate‐buffered saline (PBS) twice. All experiments were repeated three times, and an inverted microscope was used to enumerate and visualise the cells [45].

### 2.4 MTT assay

Cells (1 × 10^4^) were seeded in each of the wells of a 96‐well plate for 48 h (5% CO_2_, 37°C). After reaching a 70% confluence, and at 24 h after the treatment, an MTT reagent (10 *μl*, at a final concentration of 0.5 mg/ml) was added to each well and incubated for 4 h. Then acidified isopropanol, as an MTT destaining solution (100 *μl*), was added for 15 min while shaking the solution. Finally, 570 nm optical density was read using a microplate reader (Epoch, Biotek). After analysis in IBM® SPSS® Statistics (SPSS) software, the 50% maximal inhibitory concentration (IC_50_) was determined [45].

### 2.5 Cell apoptosis assessment using acridine orange and propidium iodide

In this method, acridine orange and propidium iodide fluorescent dyes were used. For staining, the cells (a 5‐ml suspension) were cultured in flasks and incubated for 24 h. Afterwards, the culture medium was drained and the cells were treated with different concentrations of the SLNs for 48 h. After incubation, the cells were detached from the bottom of the flask using trypsin and centrifuged at 2700 rpm for 5 min. The supernatant was then removed and the cell pellet was diluted with 1 ml PBS. In the next step, 10 *μl* of the cell suspension was mixed with acridine orange (10 *μl*) and propidium iodide (10 *μl*) and incubated for 5 min. Then the resultant mixture (30 *μl*) was poured on a slide and another slide was placed on it. Finally, the slides were photographed and examined by using a fluorescent microscope (XDS‐3FL/3FL4, OPTICA) [46].

### 2.6 Apoptosis and cell‐cycle assessments using flow cytometry

Flow cytometry was performed according to a standard method [47]. MCF‐7 cells were cultured and treated with different concentrations of the SLNs for 48 h. Afterwards, the cells were trypsinised and washed with PBS twice and then fixed with cold ethanol (70%) for 2 h. After completely removing the alcohol, the cells were collected and washed with PBS at 1200 rpm for 3 min. The cells were preserved in PBS containing 50 *μg*/ml of RNase A for 2 h at 37°C; finally, they were mixed with 25 *μg*/ml of PI stain and analysed using a flow cytometer (BD FACSCalibur) according to the manufacturer's guidelines.

### 2.7 Morphological assessments

The effects of different concentrations (0, 45, 90 and 180 *µg*/ml) of the fabricated SLNs on MCF‐7 cancer cells’ morphology was evaluated by using an inverted light microscope at 200× magnification. For this purpose, 5 × 10^5^ cells were seeded in each of the wells of a 6‐well plate and exposed to the SLNs. After 48 h of exposure, the morphological features were observed [48].

### 2.8 Statistical analysis

In SPSS (SPSS Software products, Marketing Department, SPSS) software, the data was analysed using the ANOVA test and GLM procedure. The Duncan's multiple range test was applied to determine statistical significance at a *P* value of <0.05. To designate P values of <0.05, <0.01, and <0.001, one‐, two‐, and three‐star markers were used. All experiments were done in triplicate, and the results were expressed as mean ± standard deviation.

## 3 RESULTS AND DISCUSSION

### 3.1 Characterisation of the biosynthesised SLNs

**FIGURE 1:**
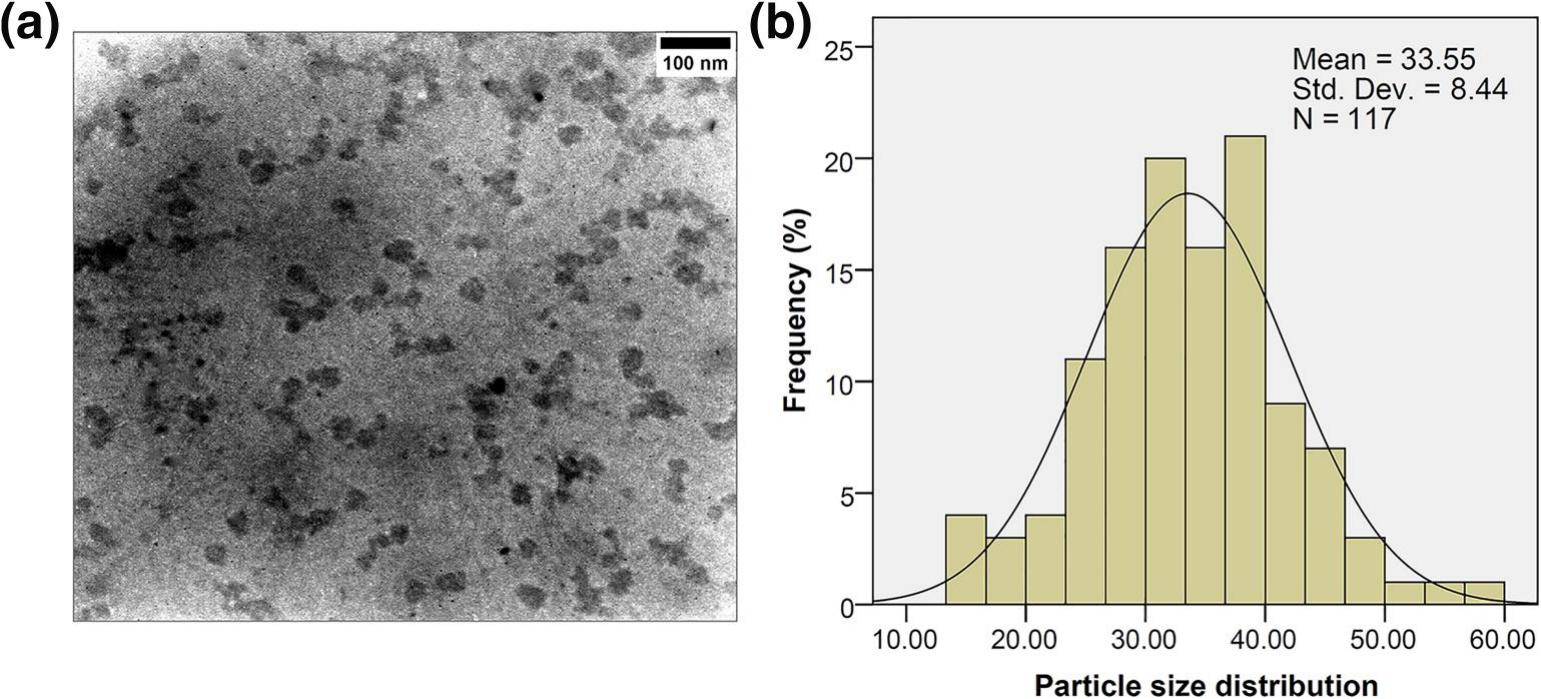
(a) Transmission electron microscopyimage of the prepared solid lipid nanoparticles (SLNs), (b) SLNs’ dimensions and dispersion in the solid form

TEM, which uses focussed beams of electrons to record a picture, can magnify biological samples by about 30 million times and is a very powerful tool for understanding the exact size and morphology of SLNs (Figure 1). The synthesised particles displayed a low tendency for aggregation and good integrity. Most of the SLNs had sizes between 14 and 56 nm, indicating a narrow size distribution. The results showed that the SLNs had spherical and hemispherical shapes and represented a mean size of 33.55 ± 8.44 nm in their solid forms.

### 3.2 Dynamic light scattering and zeta potential

**FIGURE 2:**
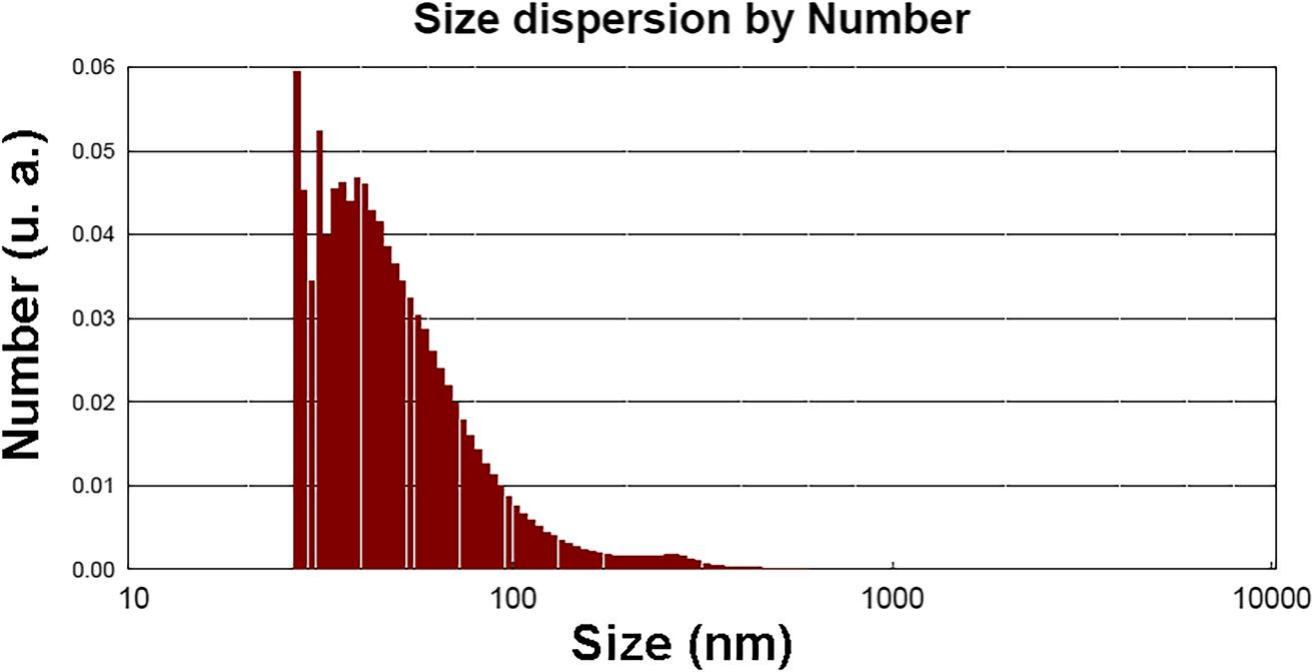
The size dispersion of the prepared solid lipid nanoparticles

The DLS technique measures the Brownian motion to determine the hydrodynamic sizes of NPs. In our study, DLS analysis revealed the particles as spheres consisting of a core particle (the prepared SLN) surrounded by surface‐bound moieties such as ions and water molecules (Figure 2). The hydrodynamic size of the SLNs was about 55.43 nm with a polydispersity index of 0.37. These findings were in agreement with our TEM observations. Our results showed that the mean particle size and, subsequently, the hydrodynamic size increased secondary to the interactions between the SLNs and the bound water molecules and ions. Surface charge analysis illustrated negatively charged SLNs with a zeta potential of −29.54 ± 11.67 mV. As our results indicated, the SLNs could repel one another due to the negative net surface charge, preventing the particles from forming aggregations in the solution. This was also confirmed by the TEM analysis.

### 3.3 SLNs toxicity against the MCF‐7 breast cancer cell line

**FIGURE 3:**
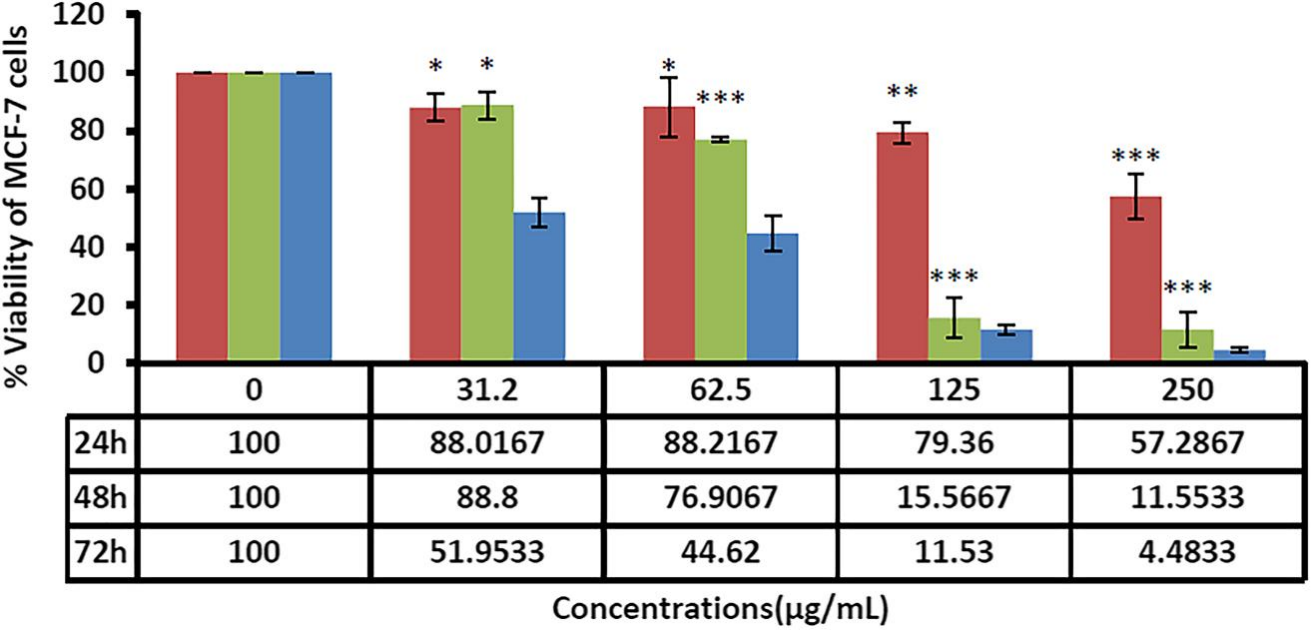
The toxicity of the fabricated solid lipid nanoparticles against the MCF‐7 breast cancer cell line at different concentrations and times. Results have been presented as mean ± standard deviation (n = 3), ***; P < 0.001, **; P < 0.01, *; P < 0.05

**FIGURE 4:**
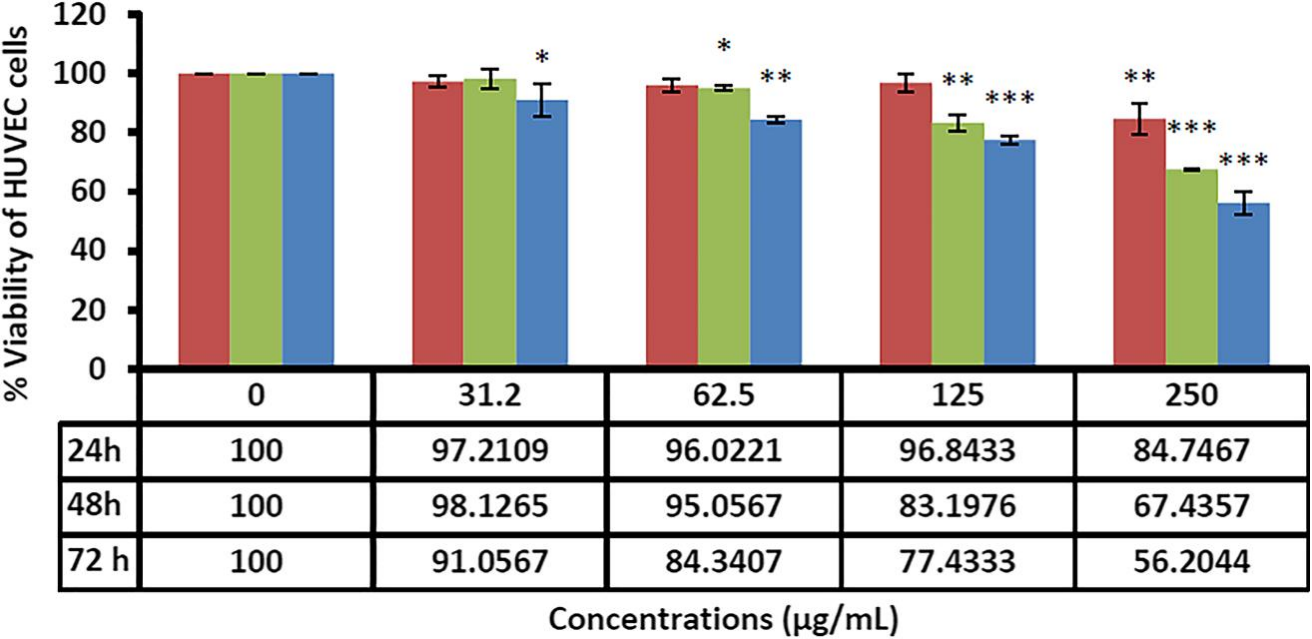
The toxicity of the fabricated solid lipid nanoparticles against normal human umbilical vein endothelial cells at different concentrations and times. Results have been presented as mean ± SD (n = 3), ***; P < 0.001, **; P < 0.01, *; P < 0.05

**FIGURE 5:**
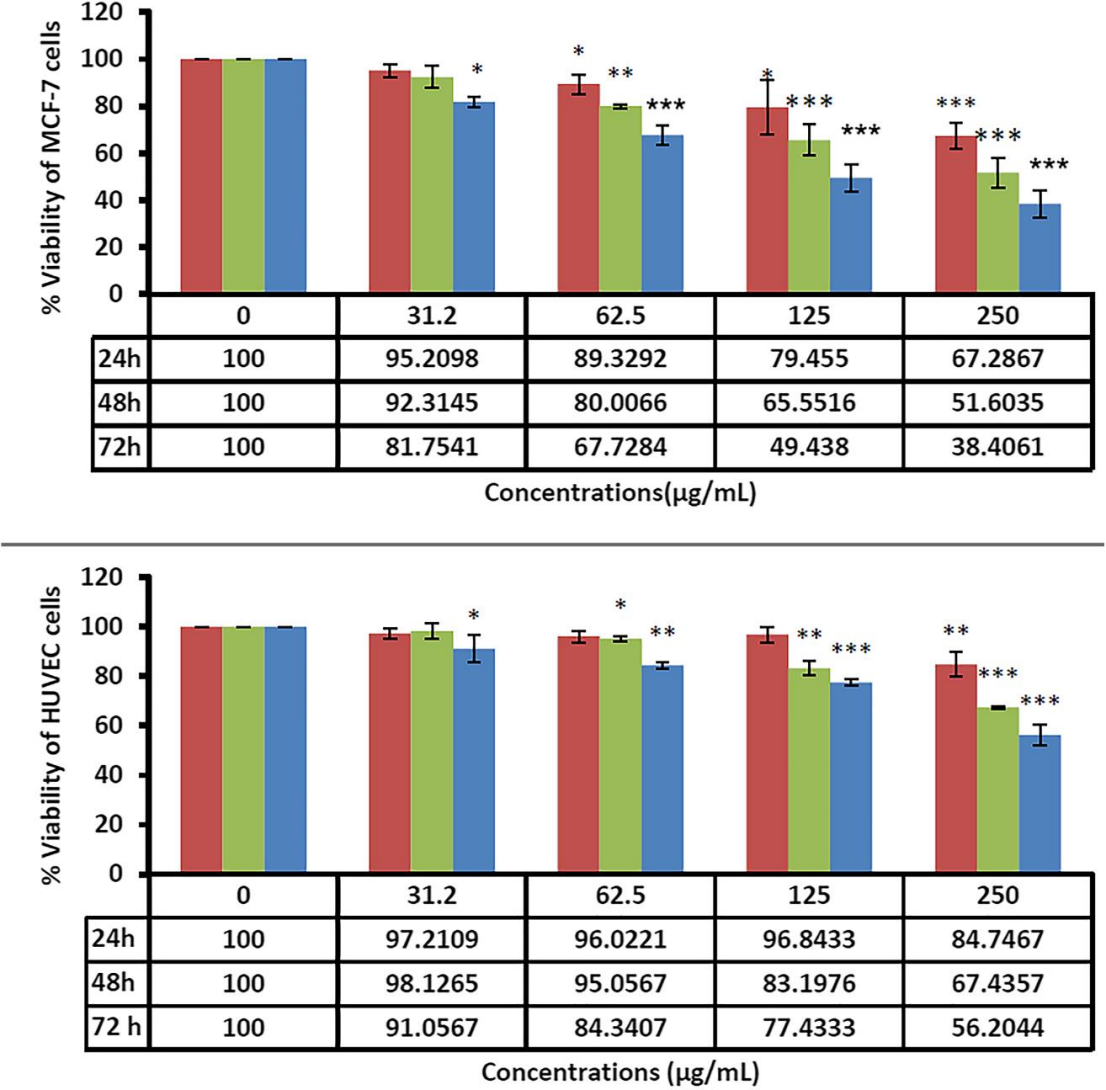
The toxicity of *F. vulgare* oil against normal human umbilical vein endothelial cells and cancerous MCF‐7 cells at different concentrations and times. Results have been presented as mean ± standard deviation (n = 3), ***; P < 0.001, **; P < 0.01, *; P < 0.05

Breast cancer is the most common neoplastic disease and the leading cause of cancer‐related death among women worldwide [49, 50]. In recent times, nanotechnology has shared a significant contribution to cancer treatment [51, 52]. Nano‐based strategies have boosted the effectiveness of drug delivery and molecular targeting approaches in parallel with a reduction in their side effects [53, 54]. Nano‐biomaterials seem to be promising key mediators for overcoming many problems in cancer treatment [55]. SLNs are well‐known nanomaterials that have now presented the maximum level of commercialisation [56, 57]. SLNs can be used to deliver numerous hydrophilic and lipophilic therapeutic factors. These NPs offer no residual contamination and prolonged physical stability, are inexpensive, and can be produced on a large scale [58]. Solid NPs, either alone or in combination with loaded therapeutics such as doxorubicin and ascorbic acid, can induce apoptosis in tumour cells or boost the effectiveness of conventional therapies [59, 60, 61]. Our results also exhibited that the synthesised SLNs induced dose‐ and time‐dependent toxicity and apoptosis against MCF‐7 cancer cells. As shown in Figure 3, the fabricated SLNs had the highest lethal effects on the cancer cells at the concentrations of 250 *µg*/ml for 72 h. Figure 4 also shows the effects of the fabricated SLNs on normal HUVEC. The IC_50_ values of the SLNs against MCF‐7 cancer cells were 89.91 and 39.49 *µg*/ml at 48 and 72 h, respectively. On the other side, the IC_50_ values for normal cells (HUVEC) were 189.16 and 189.08 *µg*/ml at 48 and 72 h, respectively. As shown in Figure 5, *F. vulgare* oil showed low toxicity against the normal and cancerous cells. MCF‐7 cells were susceptible to different doses of fennel oil with an IC_50_ of 220 ± 5.7 *µg*/ml after 48 h of treatment. The cytotoxic effect of fennel oil (IC50) on HUVEC cells were more than 250 *µg*/ml. The spherical shape and small size of the biosynthesised SLNs followed a narrow normal distribution pattern that was similar to that observed in previous investigations on SLNs [62]. Since the outer layer of the cell membrane usually has positively charged ions, the negative zeta potential of the SLNs could play a significant role in their internalisation into cells [63]. Based on the results of the present research, the toxic effects of SLNs were more pronounced against the cancer than normal cells, which could be due to the different cellular and metabolic needs of normal and cancer cells [24].

### 3.4 Apoptosis‐inducing effects of SLNs using flow cytometry

Anti‐cancer drugs promote their effects in part by inhibiting the proliferation of cancer cells by inducing apoptosis and arresting cell cycle progression. The fabricated SLNs showed anti‐proliferative effects against the cancerous cells by inducing the de‐regulation of the cell cycle (at either the G2/M or G1/S phase), as evidenced by flow cytometry. The cell cycle arrest inhibited the proliferation of cancerous cells. Also, treatment with the essential oil suppressed the progression of the cell cycle of MCF‐7 cells at the G2/M and S phases and induced apoptosis after 24 h. Various anti‐cancer agents can suppress the cell cycle at either the G0/G1, S, and/or G2/M phase and subsequently trigger apoptosis. We analysed the cell cycle to investigate the growth inhibitory effects of SLNs against the MCF‐7 cancer cell line by flow cytometry, showing that the synthesised SLNs promoted a dose‐dependent inhibitory effect on the S phase of the cell cycle. The apoptotic and cell‐cycle inhibitory effects of the SLNs could be key mechanisms through which these NPs affected the cellular growth of cancer cells [64].

**FIGURE 6:**
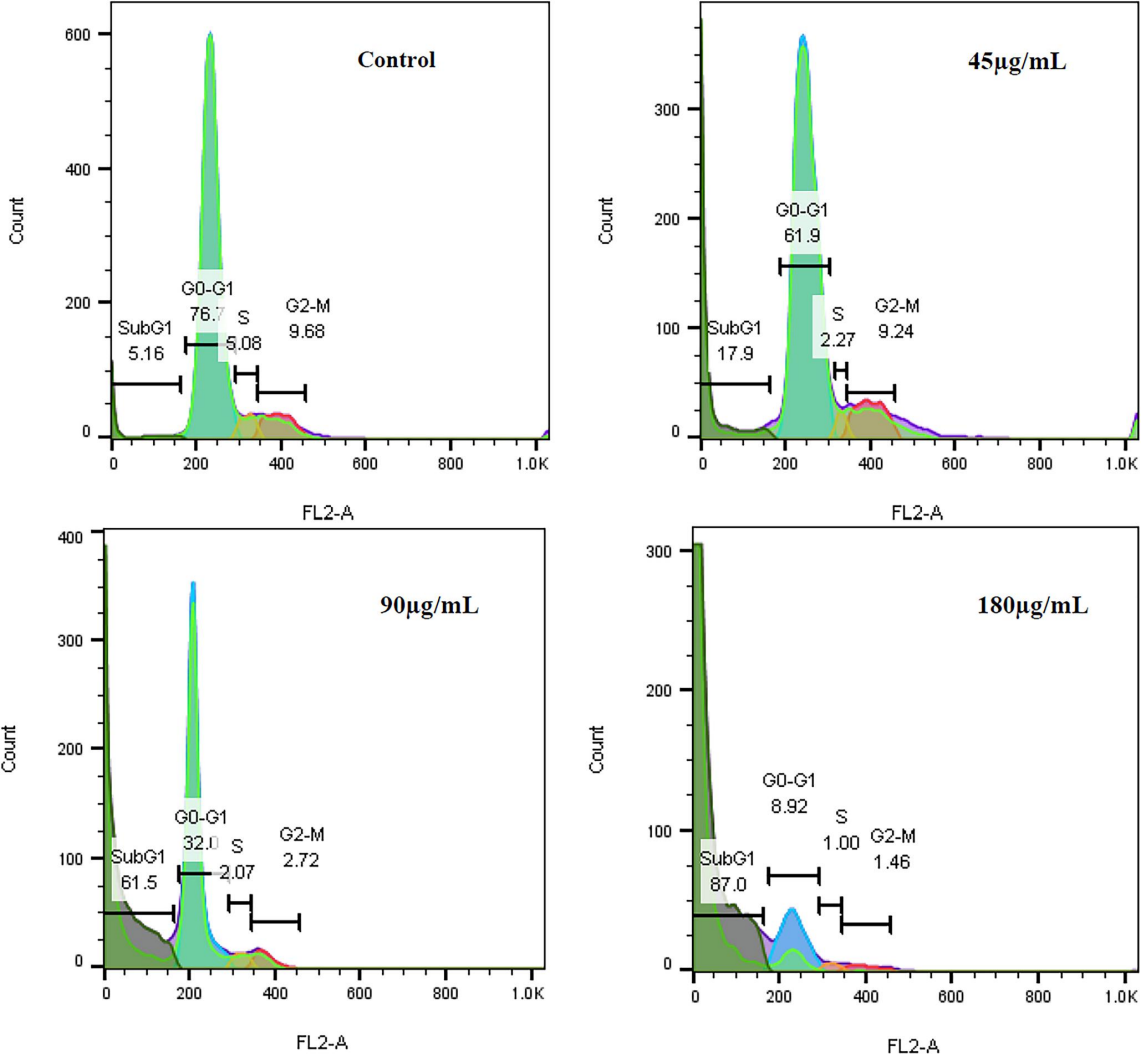
Flow cytometry analysis of apoptosis and cell cycle showed a dose‐dependent apoptotic effect for the solid lipid nanoparticles

Flow cytometry is a procedure employed to identify and measure the physicochemical features of cells and particles [65, 66]. In the present study, the results of flow cytometry (Figure 6) showed a remarkable rise in the subG1 phase of the cell cycle in the cells treated with the SLNs compared to the control. This indicated an increased rate of apoptosis in the cells exposed to the SLNs. Further, on increasing the concentration of the SLNs, the subG1 peak also intensified. At the concentration of 180 *μg*/ml, the rate of apoptotic cells remarkably increased compared with lower concentrations, indicating a dose‐dependent effect.

Cell‐cycle checkpoints are essential to regulate cellular proliferation and protect genomic stability and integrity by preventing premature cell division [67]. According to our findings, the synthesised SLNs promoted their anti‐proliferative effects partly by disrupting the cell cycle, particularly at the S‐phase, and hindering the entry of cells into the proliferative phase. The S‐phase transition is a key checkpoint regulating the proliferation of MCF‐7 cancer cells. Cell cycle arrest at the S phase, along with continuous E2F‐1 activity could be involved in apoptosis induction and the selective killing of transformed cells [68, 69]. In this regard, the induction of apoptosis and cell‐cycle arrest in cells following treatment with SLN fennel oil indicated that the G2/M checkpoint could be a possible target for treating cancer by suppressing DNA repair and cellular division. Therefore, the cells with a disrupted G2/M checkpoint may still be able to progress towards DNA replication and mitosis, which eventually lead to apoptosis. So, drugs can promote their toxicity to some extent by enforcing cell‐cycle arrest at this checkpoint. On the other hand, the entry of cells into the S phase is governed by a retinoblastoma gene product (Rb) [70]. Accordingly, hypophosphorylated Rb interacts with E2F transcription factors. The inactivation of the Rb by being phosphorylated via the act of cyclin‐dependent kinases (CDK) 2‐, CDK4‐, or CDK6 leads to the release of E2F transcription factors and progression into the S phase [71]. So, the progression of apoptosis and cell cycle arrest in SLNs‐treated cells indicated the importance of the G2/M checkpoint as a possible target for treating cancers and inhibiting cellular proliferation and DNA repair [72].

### 3.5 Apoptotic‐inducing effects of SLNs using the acridine orange/propidium iodide test

Acridine orange is a biological material employed as a fluorescent dye for analysing the cell cycle. Acridine orange penetrates into cells, which permits the dye to contact with the DNA/RNA. Acridines are extensively used in cancer research because they exclusively and preferably gather into invasive tumour cells and tissues and emit high fluorescent signals [73, 74].

**FIGURE 7:**
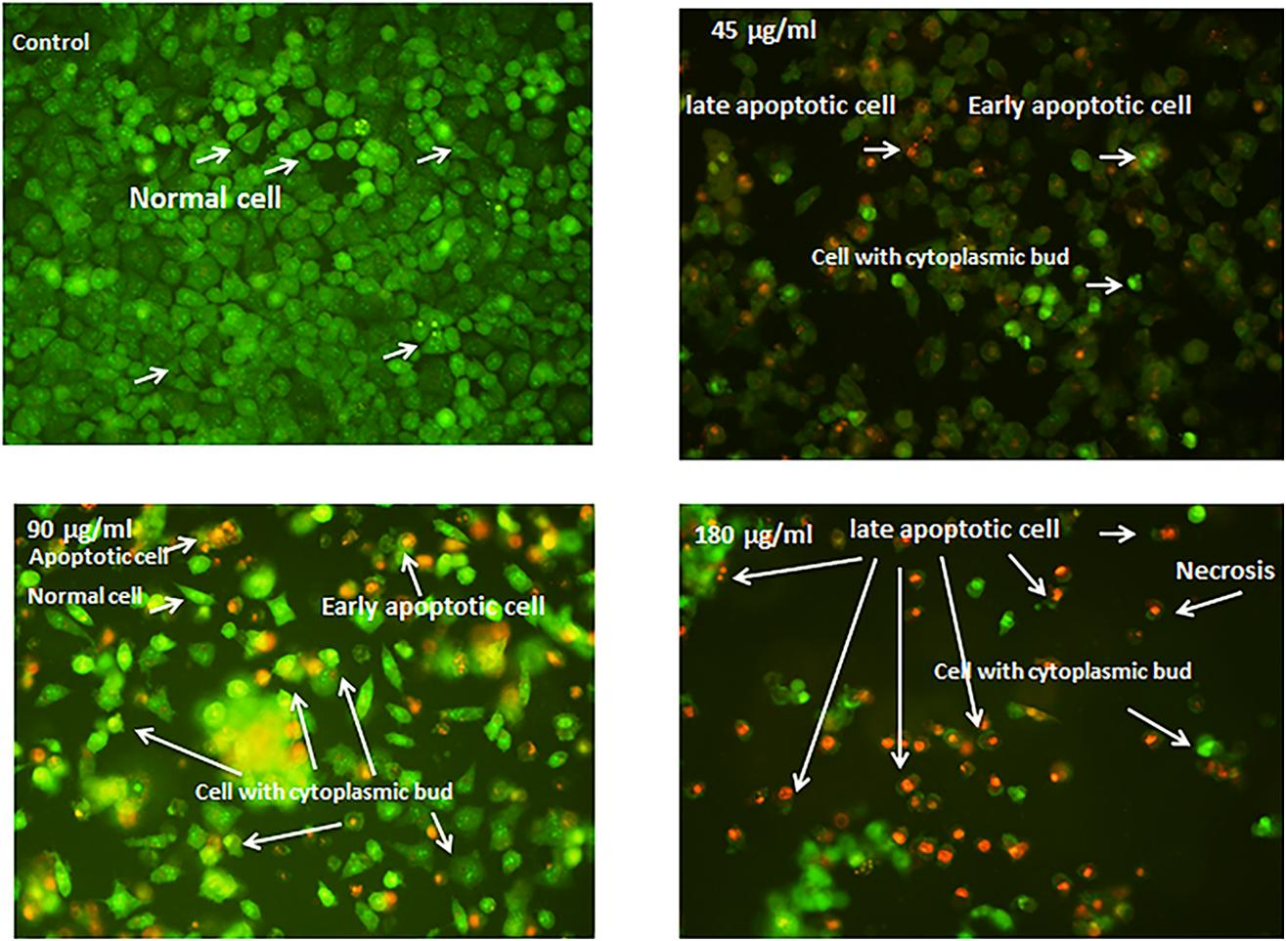
Acridine orange/propidium iodide (AO/PI) fluorescent labelling of MCF‐7 cells. MCF‐7 cells were treated with different concentrations of solid lipid nanoparticles (SLNs) and after 24 h of incubation, the cells were labelled by AO/PI and observed immediately under a fluorescent microscope at 200× magnification. Dose‐dependent reduction in viability and increase in apoptosis were observed for the SLN. Morphological alterations in the SLN‐treated cells, such as cytoplasm budding and membrane blebbing, indicated the induction of apoptosis. Some necrotic cells were also observed in higher concentrations of the SLNs

In our study, fluorescence microscopy analysis indicated predominant green‐stained (i.e. alive) normal cells. In contrast, in the cancer cells treated with the SLNs, the cells were observed to be orange to brownish as a result of the internalisation of the dye, indicating apoptotic cells (Figure 7).

For acridine orange/propidium iodide (AO/PI) fluorescent analysis of MCF‐7 cells, the cells were treated by different concentrations of the synthesised SLNs for 24 h and then labelled by AO/PI before being examined under a fluorescent microscope at 200× magnification. The fabricated SLNs decreased the cells’ viability and increased cell death dose dependently. Morphological alterations in the treated cells, such as cytoplasm budding and membrane blebbing, were observed, indicating apoptosis. Some necrotic cells were also observed on exposure to higher concentrations of the SLNs.

In the present study, the AO/PI fluorescent staining showed that the fabricated SLNs had dose‐dependent and significant (p < 0.05) cytotoxic and apoptotic effects. The apoptotic effects were supported by morphological changes such as cellular shrinkage, nuclear fragmentation, chromatin condensation, and membrane blebbing, as evidenced by the AO/PI fluorescent microscopic imaging [75]. Apoptosis in cells generally correlates with the upregulation of apoptosis‐associated genes (e.g. miR‐92a, miR‐21, Bcl‐2, caspase‐3, and p53) [76].

### 3.6 The morphology and viability of SLNs‐treated MCF‐7 cells

**FIGURE 8:**
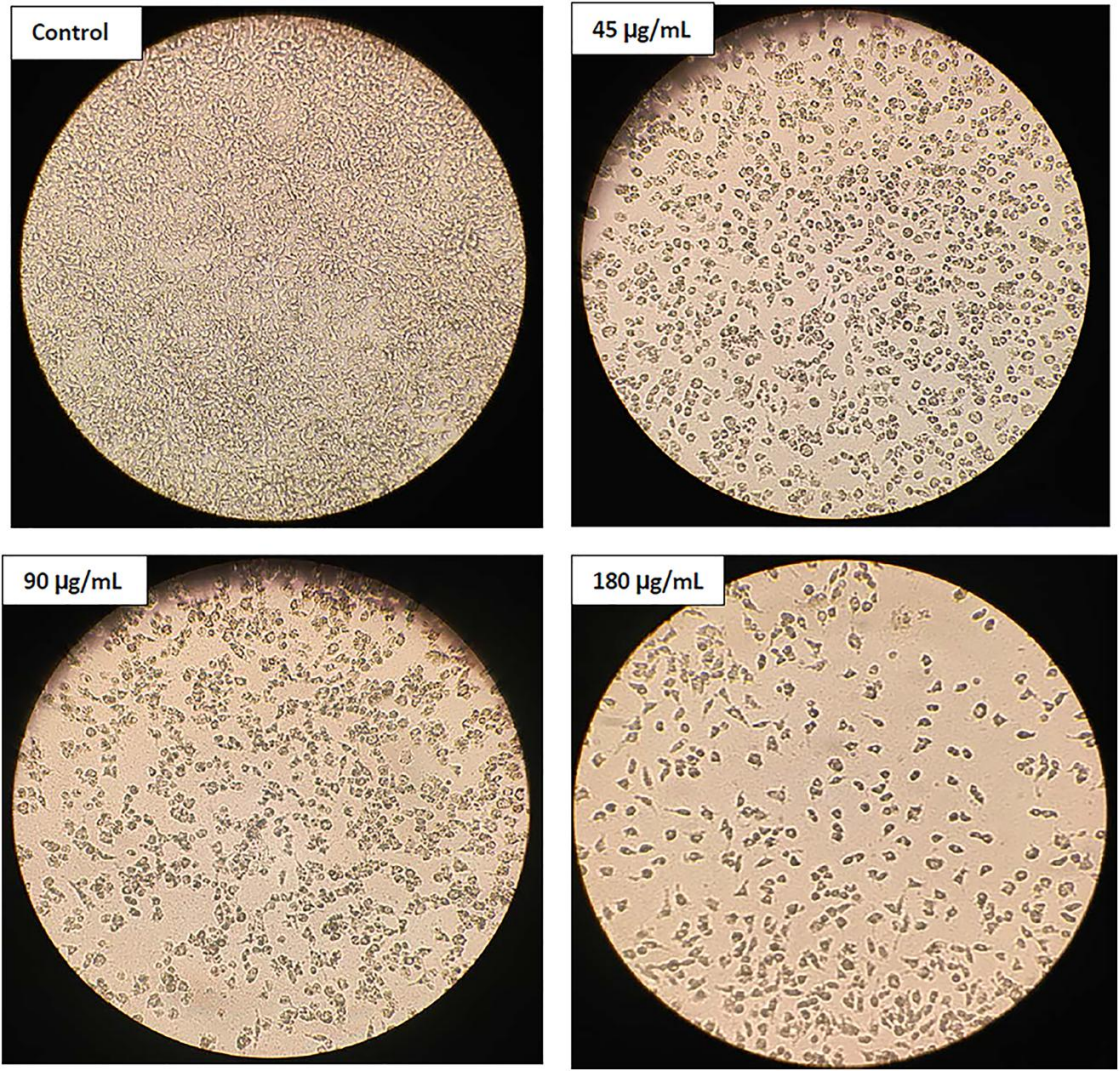
The morphological changes of the MCF‐7 cells treated with solid lipid nanoparticles at 45, 90, and 180 *μg*/ml concentrations compared to the control group. The changes were seen under an inverted light microscope (Olympus) (200× magnification)

**FIGURE 9:**
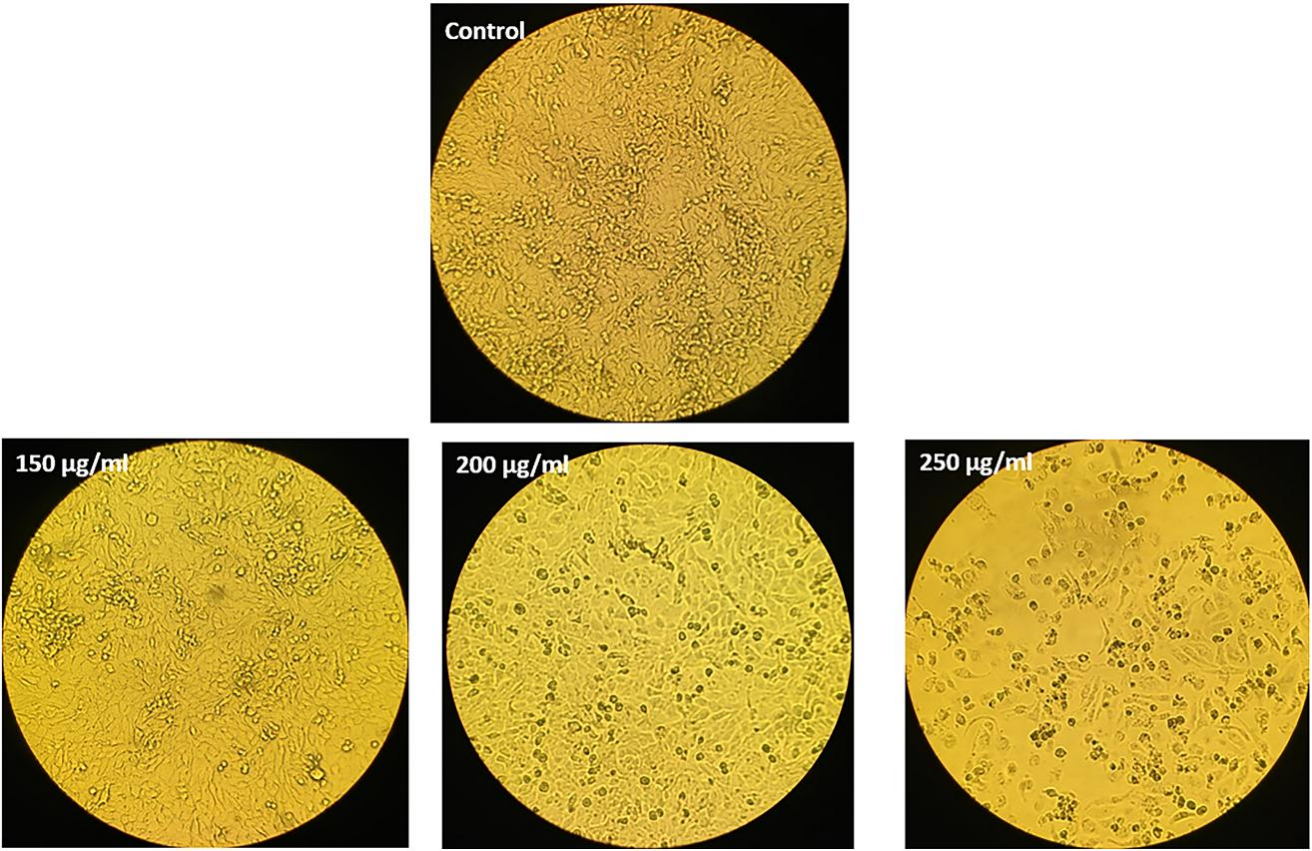
The morphology of the normal HUVEC cells treated with different concentrations of solid lipid nanoparticles. The altration were seen under an inverted light microscope (Olympus) (200× magnification)

The morphological evaluation of the MCF‐7 cells treated with the SLNs showed that by increasing the concentration of the NPs, the viability of cells decreased compared to the control, indicating dose‐dependent toxicity (Figures 8 and 9). The low cell toxicity of the SLNs could be attributed to lecithin and other components used in the aqueous phase [77]. Using SLNs can offer target‐specific and sustained release of drugs [78]. The MCF‐7 cells treated with the SLNs at the concentrations of 45, 90, and 180 *μg*/ml showed more morphological alterations and decreased cellular dimensions compared to the control group.

## 4 CONCLUSION

The aim of this study was to fabricate SLNs, as anti‐cancer agents, using a green method. For this purpose, SLNs were prepared using homogenisation and ultrasound methods. Our results showed that the SLNs had a narrow particle size distribution with an average size of 33.55 nm in the solid phase and the mean hydrodynamic size of 55.43 nm. Therefore, it seems that this method was successful in synthesising SLNs with sizes under 60 nm. The prepared SLNs delivered the highest cytotoxic effects against MCF‐7 breast cancer cells at the concentration of 250 *µg*/ml upon 72 h of exposure. The SLNs showed low toxicity against normal HUVEC cells. The acridine‐orange test and flow‐cytometry analysis demonstrated dose‐dependent cytotoxic and growth‐inhibitory effects of the SLNs against the cancerous cells. In conclusion, the prepared SLNs seemed to be utilisable as beneficial and promising chemotherapeutics for treating cancers.

## CONFLICT OF INTEREST

There is no conflict of interest to declare.
